# Angiosarcome mammaire radio-induit: à propos d’un cas

**DOI:** 10.11604/pamj.2020.36.29.21599

**Published:** 2020-05-21

**Authors:** Amine Majdoubi, Badr Serji, Tijani El Harroudi

**Affiliations:** 1 Service de Chirurgie Oncologique, Centre d’Oncologie Hassane II, Centre Hospitalier Universitaire Mohammed VI, Oujda, Maroc

**Keywords:** Angiosarcome mammaire, radiothérapie, tumeur radio-induite, Breast angiosarcoma, radiotherapy, radiation-induced tumor

## Abstract

L’angiosarcome mammaire est une tumeur conjonctive rare d’origine endothéliale vasculaire, primitive chez les patientes jeunes, radio-induite chez les plus âgées, caractérisée par sa malignité et par sa présentation clinique et radiologique polymorphe, elle est de très mauvais pronostic, par la survenue fréquente de métastases viscérales et de récidive rapide. Nous rapportons un cas d’angiosarcome mammaire, chez une patiente âgée de 43 ans, ayant un antécédent de cancer de sein, traité par chirurgie conservatrice et radiothérapie adjuvante. Nous discuterons à travers cette observation, les aspects épidémiologiques, diagnostiques et thérapeutiques de ce type de rares tumeurs agressives.

## Abstract (en)

Breast angiosarcoma is a rare vascular endothelial connective tissue tumor. It is primitive in young patients and radio-induced in older subjects. It is a malignant tumor and it manifests with polymorphic clinical and radiological features. Its prognosis is poor due to the frequent occurrence of visceral metastases and fast recurrence. We here report a case of breast angiosarcoma in a female patient aged 43 years, with a history of breast cancer, treated by conservative surgery and adjuvant radiotherapy. The purpose of this study is to highlight the epidemiological diagnostic and therapeutic features of these rare aggressive tumors.

## Introduction

L’angiosarcome mammaire (ASM) est une tumeur mésenchymateuse maligne rare, qui se développe au dépend du tissu vasculaire mammaire. Elle représente 0,004 à 1% de l’ensemble des tumeurs malignes du sein [1], et 8 à 10% des sarcomes mammaires [2]. Elle peut être primitive chez une femme jeune de 40 ans, ou radio-induite chez une femme plus âgée ayant bénéficiée d’un traitement conservateur pour un cancer du sein, incluant une chirurgie conservatrice et une radiothérapie adjuvante [3]. L’incidence des angiosarcomes secondaires ne cesse d’augmenter, compte tenu du recours au traitement conservateur locorégional. Ils touchent généralement la peau, rarement la paroi thoracique ou le parenchyme mammaire [4]. Son diagnostic de certitude est purement histologique, affirmant le caractère vasculaire des angiosarcomes qui expriment typiquement et spécifiquement les marqueurs endothéliaux CD31 et CD43 [5]. Nous rapportons l'observation d’un angiosarcome radio-induit chez une patiente âgée de 43 ans ayant bénéficié d’un traitement conservateur pour un cancer du sein il y a 6 ans. A travers cette dernière nous discuterons les aspects épidémiologiques, diagnostiques, thérapeutiques et évolutifs de ce type de tumeurs.

## Patient et observation

**Figure 1:**
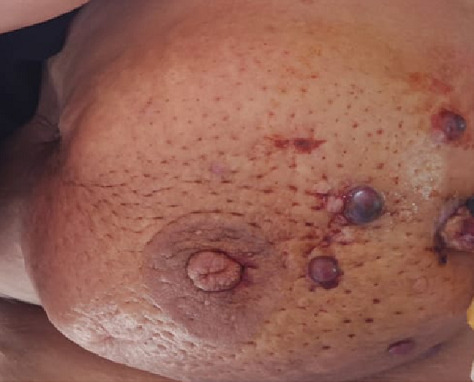
Nodules de permeation violacés avec des signes inflammatoires en regard

**Figure 2:**
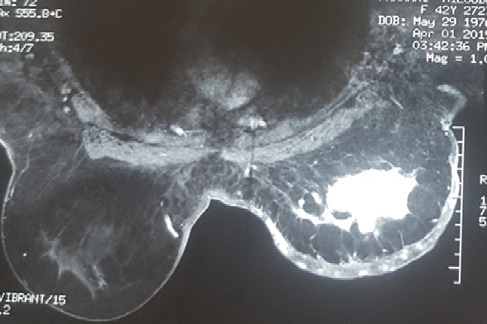
Aspect IRM d’une masse intra-mammaire droite, spéculée, mal limitée, de contours irréguliers, qui se rehausse précocement et de façon intense après injection du PDC associée à des lésions satellites

**Figure 3:**
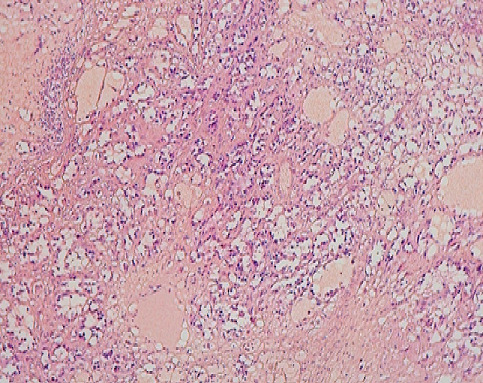
Microphotographie montrant une prolifération fusocellulaire avec formation de canaux vasculaires le plus souvent anastomosés (HE; 100X)

**Figure 4:**
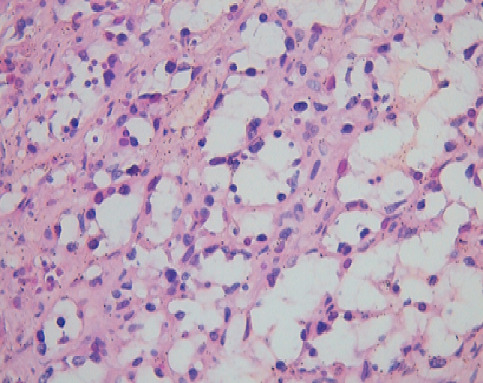
Microphotographie à plus fort grossissement montrant l’aspect atypique des cellules endothéliales (HE; 400X)

Il s’agit d’une patiente âgée de 43 ans, 3^ème^ geste, 3^ème^ pare, qui avait présenté, à l’âge de 36 ans, un carcinome canalaire infiltrant grade II de Scraff et Bloom , à cheval des quadrants externes de 2,5 cm avec un bilan d’extension négatif (T2N2M0), pour lequel elle avait bénéficié d’une tumorectomie avec curage axillaire en premier temps (limites d’exérèse saines, RE +, RP +, HER (-), 4 N+/20 N avec effraction capsulaire), puis en second 6 cures de chimiothérapie [3 séances AC60 (Adriamycine/ Cyclo-phosphamide), puis 3 séances de taxane] , puis pour clôturer le traitement de la tumeur, une radiothérapie externe a été indiquée (50 Gy sur la glande mammaire, chaine mammaire interne et sur la région sus Clavière droite avec surimpression de 15 Gy au niveau du lit tumoral). Une hormonothérapie anti-oestrogénique a été prescrite, du fait de la positivité des récepteurs hormonaux, pour une durée de 5 ans. La patiente a été suivie régulièrement. Six ans après le traitement locorégional conservateur, la patiente a consulté pour une mastodynie, avec des signes inflammatoires au niveau du sein irradié. L’examen clinique trouve au niveau du sein irradié un nodule fixe du quadrant infero-externe de 3 cm avec des signes inflammatoires en regard, et des nodules de permeation cutanées violacés (Figure 1). Une mammographie a retrouvé une masse solido-kystique en faveur d’une image de cytostéatonécrose en regard de la cicatrice. Une IRM mammaire, a objectivé l’existence, d’une masse mammaire droite de 6 cm fortement suspecte, une biopsie écho-guidée a retenu le diagnostic d’angiosarcome. Le bilan d’extension n’a pas trouvé de lésions secondaires à distance (Figure 2). Une mastectomie radicale a été réalisée, dont l’analyse anatomopathologique (Figure 3, Figure 4) a confirmé le diagnostic d’angiosarcome mammaire de haut grade selon le grading de Donnel (Figure 3, Figure 4), la taille de la tumeur était de 6 *3,5 cm, infiltrant la peau et le parenchyme mammaire sous-jacent. Les marges de résection étaient supérieures à 3 cm. Les suites post-opératoires ont été simples, vu la taille de la tumeur, une chimiothérapie adjuvante a été indiquée à base d’antracycline et d’isofosfamide lors d’un staff d’une Réunion de Concertation Pluridisciplinaire. Actuellement la patiente est toujours sous chimiothérapie, elle est suivie régulièrement dans notre formation.

## Discussion

Le traitement conservateur des cancers du sein demeure une option thérapeutique de choix grâce à la généralisation du dépistage et le diagnostic précoce des lésions cancéreuses. La radiothérapie constitue une étape obligatoire dans le traitement conservateur du cancer du sein, elle est souvent indiquée en post opératoire. Or, une dose cumulée supérieur à 40 Gy, peut induire à long terme le développement de certaines tumeurs comme l’angiosarcome [6]. L’angiosarcome est une tumeur maligne rare, caractérisée par la néoformation des canaux veineux au niveau des tissus cutanés et sous-cutanés, ils siègent préférentiellement, au niveau de la tête, des membres. Le sein représente 9 % de l’ensemble des localisations [7]. C’est une tumeur qui touche primitivement la femme jeune en période d’activité génitale, surtout entre 30 et 40 ans [1]. Mais, toutes les tranches d'âge sont concernées, de 13 à 85 ans.

L’homme est rarement concerné, de notre connaissance seulement neuf cas ont été décrit dans la littérature. (Pructer 1958, Schakerford 1968, Yadav 1976, Rainwater 1986, Mansouri 2000, Garnier G 2005, WR Bouraoui 2011, Da Silva 2018, Nikolaos Tsapralis 2019). Plusieurs facteurs ont été incriminés dans la genèse des angiosarcomes [8]: a) l’exposition l’arsenic, au chloroéthène, pour les formes hépatiques; b) l’irritation chronique endoluminale par un corps étranger pour les localisations digestives; c) un lymphœdème chronique (syndrome de Stewart-Treves) pour les angiomes des membres; d) une radiothérapie préexistante permet d’expliquer les formes secondaires.

La forme radio-induite survient, surtout, chez la femme âgée, avec un temps de latence de 29 à 72 mois [1], ce qui est le cas pour notre patiente. L’ASM est découverte dans la majorité des cas, suite à un nodule ayant un caractère vasculaire, pulsatile, de couleur noir /violacé, volumineux avec une taille souvent comprise entre 2 et 11 cm [2], qui augmente rapidement de taille. Ces caractères répondent à la description sémiologique de notre cas (un nodule noir violacé qui a doublé de volume dans 3 mois). Deux signes cliniques sont pathognomoniques de l’angiosarcome du sein: l’aspect violacé de peau en regard de la tumeur et le caractère pulsatile de la masse [9]. Les adénopathies axillaires sont exceptionnelles, elles ne concernent que les formes très évoluées [10]. Les travaux de CAHAN ont permis d’établir des critères diagnostiques pour l’angiosarcome radio-induit [11], qui sont basés sur les items suivants: a) un antécédent d’irradiation mammaire; b) une période latence de plusieurs années (plus de cinq ans); c) la survenue du sarcome dans le champ irradié; d) la confirmation histologique de l’origine sarcomateuse.

Notre observation a répondu à ces critères diagnostiques. La mammographie n’est pas spécifique, elle retrouve souvent une image d’asymétrie focale de densité [12]. La tumeur est souvent difficilement visible chez des femmes jeunes aux seins denses. À l’échographie, l’ASM se manifeste comme une image hyperéchogène, hypoéchogène ou mixte, simulant une masse tissulaire, hétérogène avec zones nécrotiques et hémorragiques au centre [13]. L’étude au Doppler confirme l’origine vasculaire de la tumeur, en montrant une hyper vascularisation intense [5]. L’apport de la TDM est crucial, elle montre une masse hypervasculaire qui se rehausse intempestivement au temps artériel avec une homogénéisation partielle au temps portal [14]. La tomodensitométrie trouve surtout sa place pour rechercher les métastases à distance. L’IRM retrouve une masse hétérogène avec des zones hémorragiques ou des lacs veineux en hypersignal T1, l’intensité du rehaussement dépond du grade histologique, ainsi un angiosarcome de haut grade qui se rehausse de façon intense avec wash-out rapide [14]. L’intérêt de l’IRM se résume dans l’évaluation de l’extension locale de la tumeur, notamment l’extension vers le plan musculaire sous-jacent [5]. Le diagnostic de certitude est purement histologique, les angiosarcomes se comportent macroscopiquement comme une tumeur agressive localement, infiltrantes contenant des zones nécrotico-hémorragique. Microscopiquement, les angiosarcomes présentent des anomalies structurelles en fonction de leurs stade de différenciation, un angiosarcome bien différencié infiltre le derme, l’hypoderme ou le parenchyme par canaux vasculaires néoformés, qui détruisent les fibres du tissu conjonctif sans détruire les canaux galactophoriques ou les lobes mammaires [15]. L’agressivité des lésions est proportionnelle au bouleversement de l’architecture des cellules endothéliales, un angiosarcome peu différencié est composé d’un tissu pluristratifié de cellules endothéliales qui présentent des anomalies nucléaires, des projections intra-luminales papillaires et des inclusions anormales des hématies dans le cytoplasme [16]. Les tumeurs peu différenciées réalisent un aspect de tumeur épithéliale contenant des zones de nécrose et de l’hémorragie.

Au terme de ces critères histologiques, deux classifications histologiques ont été proposées par Donnel [17], et par Merino [18]. Chacune de ces classifications comporte trois types histologiques reprenant les mêmes items, La plus utilisée actuellement est celle de Donnell (Tableau 1). Pour les tumeurs difficilement identifiables sur l’étude microscopique, la nature vasculaire de la lésion peut être confirmée par l’étude immunohistochimique. Les marqueurs endothéliaux CD31, CD34, facteur VIII, Ulex europaeus agglutinine 1 et vascular endothelial growth factor (VEGF) sont typiquement exprimés par les angiosarcomes. Seule l’expression du CD 31 est spécifique à ces derniers [5]. Le pronostic dépend du grade histologique, avec une survie à 10 ans toute forme confondue (primitive ou secondaire) à 76% pour les formes bien différenciées ou grade 1, 20% pour les grade 3 ou peu différenciées. L’angiosarcome a un mauvais pronostic avec une survie médiane de 15 ans pour les grades I, 12 ans pour les grades II et seulement de 15 mois pour les grades III. Indépendamment du grade histologique, la taille de la tumeur constitue un facteur pronostic, pour une taille de 5 cm, la survie médiane à 10 ans est réduite à 68% pour les tumeurs de grade I [5]. L’évolution naturelle de l’angiosarcome radio-induit est plus au moins rapide vers le décès qui survient dans un tableau de dissémination métastatique après une survie médiane de 24 mois [1]. La qualité du geste chirurgical est un facteur pronostic, une résection R0 doit être toujours envisagée. La mastectomie radicale constitue un traitement de choix pour ce type de tumeur, le curage axillaire n’est justifié que s’il existe un envahissement ganglionnaire [19]. Dans la littérature, la chimiothérapie trouve son intérêt dans la situation adjuvante, face à la chirurgie seule, elle améliore la survie et permet une diminution des récidives locales et des métastases. Les taxanes et les anthracycline ont une activité anti-angio-génique intéressante, avec un taux de réponse globale initiale de 20 à 60 % d’où, l’intérêt de leur utilisation en situation adjuvante [20].

**Table 1:** Grades histologiques des angiosarcomes

**Grade histologique**	Critères histologiques
**Bas grade**	Vaisseaux anastomosés
	Rares touffes endothéliales
	Quelques cellules endothéliales hyper chromatiques
	Mitoses absentes ou rares
	Absence de nécrose
**Type 2: grade intermediaire**	Aspect prédominant du grade 1
	Quelques foyers de formation papillaires
	Zones massives disposées en cellules fusiformes
	Représentant moins de 20% de la tumeur
	Atypies cellulaires et mitoses
	Absence de nécrose
**Type 3: haut grade**	Prédominance de zone massive à cellules fusiformes
	Atypies cellulaires marquées
	Nombreuse mitoses
	Foyer de nécrose hémorragiques
	Exceptionnelles aspect de grade 1

## Conclusion

L’angiosarcome radio-induit est une rare tumeur maligne, elle survient chez les femmes âgées ayant bénéficié d’un traitement conservateur du cancer du sein. Elle est caractérisée par son mauvais pronostic, sa tendance vers les métastases viscérales et un risque de récidive. Cliniquement, il se manifeste par l’apparition dans les champs irradiés d’une masse violacée qui augmente rapidement de volume avec des signes inflammatoires en regard. L’imagerie est peu concluante, l’imagerie par résonnance magnétique a une place cruciale pour évaluer l’extension locorégionale avant de réaliser une chirurgie curative qui doit être R0. Seule l’étude histologique et immunohistochimique permettent de retenir le diagnostic de ce type tumeur. Une chimiothérapie adjuvante, pour les formes secondaires radio-induite est toujours indiquée, elle permet de réduire le risque de récidive tumorale. Le pronostic dépend du degré de différenciation tumoral, sa taille, et de la qualité d’exérèse chirurgicale.

## Conflits d’intérêts

Les auteurs ne déclarent aucun conflit d'intérêts.
